# Levelling Education Outcomes for Students With Medical and Mental Health Needs

**DOI:** 10.5334/cie.7

**Published:** 2020-08-17

**Authors:** Leah Cave, Kirsten Hancock, Caleb Jones, Deb de Lacy, Trevor Briedis, Angelina Moffatt, Grant Wheatley

**Affiliations:** 1 Telethon Kids Institute, AU; 2 School of Special Educational Needs, Medical and Mental Health, Western Australia, AU

**Keywords:** Education, Health conditions, Evaluation, Hospital-based educators

## Abstract

Students’ educational and behavioural outcomes can be adversely impacted by the unique challenges posed by chronic health conditions. As some children and adolescents may live with these challenges throughout their education, hospital-based educators play a crucial role in reducing the impacts of health conditions on educational outcomes. This study assessed the extent to which the support provided by the School of Special Educational Needs: Medical and Mental Health (SSEN:MMH, Western Australia) attenuated the negative association between higher absences and lower student outcomes. Administrative education records relating to absences, student behaviour, achievement outcomes, and level of support provided by the SSEN:MMH were used to assess the study questions. Regression models revealed no significant association between higher levels of teaching support and student outcomes after controlling for baseline characteristics. However, the negative association between higher absences and lower academic achievement was lower among students receiving higher levels of liaison. Additional analysis highlighted challenges in evaluating student outcomes, including the finding that most students receiving support missed at least two weeks of school over a year but received less than the equivalent of two days of teaching support, suggesting that the available measures were not sensitive to the level of teaching support provided. Together, the findings of this study suggest that liaison services informing schools about the educational needs of students are an important tool for supporting students academically and that the process of supporting students with chronic health conditions is not a simple task given the varying complexity of student needs and behaviours.

## Introduction

Students with chronic health conditions can experience adverse school outcomes, including reduced school engagement and achievement and poorer peer relationships and school social functioning (Lum et al., 2017; Runions et al., 2019). These outcomes are due in part to the sporadic and occasionally prolonged periods of absence from their enrolled school (Crump et al., 2013). Specifically, routine absences from school can increase the risk of low academic achievement (Champaloux & Young, 2015; Hancock, Shepherd, Lawrence, & Zubrick, 2013), disengagement from school (Forrest, Bevans, Riley, Crespo, & Louis, 2011), social isolation (Jackson, 2013), decreased social competence (Martinez, Carter, & Legato, 2011), and psychosocial functioning (Quach & Barnett, 2015). Health conditions can also lead to reduced education and employment outcomes into adulthood, with stronger negative effects observed for mental than for physical health conditions (Hale, Bevilacqua, & Viner, 2015). With medical advancements improving the prognosis of many illnesses, the provision of educational supports for children and adolescents living with chronic illness has become increasingly relevant (Irwin & Elam, 2011).

Educational support for students with chronic health conditions can help ensure that the educational needs of these students are being addressed (Hopkins, 2016; Lum et al., 2017), in additional to meeting their rights to receive an education (United Nations, 1989). Several predominantly qualitative research projects conducted in Australia have examined the experiences and support needs of students with chronic health conditions as they navigate the education system (Crosby, Bauer, Hughes, & Sharp, 2008; De Jong & Griffiths, 2008; Hopkins, Green, Henry, Edwards, & Wong, 2014; Hopkins, Moss, Green, & Strong, 2014; Royal Children’s Hospital Research Institute, 2014; Shiu, 2004a, 2004b). In these studies, students, teachers, and parents all identified the need for open and ongoing communication between health and education systems to ensure continuity in students’ education and socioemotional development (Hopkins, Green, et al., 2014; Hopkins, Moss, et al., 2014; Royal Children’s Hospital Research Institute, 2014; Shiu, 2004a, 2004b). Professional education for teachers was also identified as a critical need, ensuring that teachers received both general information about health conditions and individual information about managing the health of students in their care (Hopkins, Green, et al., 2014; Shiu, 2004a). Finally, the research identified collaborative development of an education plan between educators and healthcare professionals as key, allowing for input from both education and health systems in managing appointments and recovery, making recommendations for transitions back to the enrolled school, and sharing information about students’ progress and ongoing needs (Hopkins, Green, et al., 2014; Shiu, 2004a, 2004b).

While many studies focused on adapting support for students within their enrolled school, hospital-based educators and hospital schools were recognised as a critical link in the chain of support for students with chronic health conditions. Qualitative evaluations of various components of hospital schools across Australia have found generally positive outcomes for students engaged in their support (Crosby et al., 2008; Hopkins, Moss, et al., 2014; Royal Children’s Hospital Research Institute, 2014). Communication was commonly cited as a critical factor in ensuring positive outcomes for students (Crosby et al., 2008; Hopkins, Moss, et al., 2014). Effective collaborative models included regular case conferences between teachers and health professionals, ongoing information sharing between all staff involved in students’ care and their families as well as provision of continuing professional learning for teachers (Crosby et al., 2008). In addition, the need for personalised education plans was again highlighted, with inquiry-based pedagogy and child-centred learning identified as important features of these plans to allow flexibility and recognise the diversity of student ages, backgrounds, health conditions and learning needs (Hopkins, Moss, et al., 2014). Finally, it was acknowledged that the role of hospital-based educational support does not end after discharge from hospital. Children and adolescents sometimes spend up to a month or more recovering at home, so there is a need for ongoing educational support in the community and at home while students transition back into their enrolled school on a full-time basis (Royal Children’s Hospital Research Institute, 2014). Hospital schools can play a critical role in this transition by offering ongoing liaison and – where relevant – in-home teaching programs (Royal Children’s Hospital Research Institute, 2014).

Beyond evidence generated by these qualitative studies, limited quantitative evidence has contributed to understandings surrounding the effectiveness of educational models that support students with chronic health conditions in Australia. In part, this gap is due to limitations surrounding the availability of quality longitudinal data collected in these settings. A previous report using administrative education records identified that students receiving support from a hospital school had lower attendance rates and grades than the broader student population, and that their attendance and grades were lower before their first contact with the hospital-based education service occurred (Hancock & Lima, 2017). However, the analysis was only descriptive and did not account for any differences that may exist between students with a higher propensity for chronic health or mental health problems, which is an important consideration given the volume of research showing socioeconomic gradients in health and mental health outcomes for children and young people (Johnson, Lawrence, Perales, Baxter, & Zubrick, 2018; Nicholson, Lucas, Berthelsen, & Wake, 2012).

Specialised data collection is required to ensure that key variables are included in analysis and that outcomes encompass not only educational attainment but also school engagement and communication between educators and healthcare providers. The current study aims to address this gap by conducting a quantitative analysis to assess patterns of service use and the association between varying levels of support from hospital-based education services and students’ educational and behavioural outcomes.

### The Current Study

This study aimed to assess how support services provided by the School of Special Educational Needs: Medical and Mental Health (SSEN:MMH, Western Australia) relate to student educational and behavioural outcomes. Given the aim of the SSEN:MMH is to provide support to students to address the educational barriers they encounter from experiencing higher absences, the supports the school provides should mitigate the adverse effects of missing school. Therefore, the two hypotheses addressed in this study were (a) consistent with the broader literature, SSEN:MMH students with a higher number of absences in year *t* will have lower achievement and behavioural outcomes in year *t+1* than SSEN:MMH students with fewer absences, and (b) the negative association between higher absences and lower outcomes will be lower among students who receive more support from the SSEN:MMH.

## Method

### Study Population

The study assessed the administrative records of all Year 4 and Year 8 SSEN:MMH students who were enrolled in a government school at any time during 2008–2016 (inclusive) in Western Australia (WA). These records were sourced from a dataset provided by the SSEN:MMH and matched to administrative education records routinely collected and held by the Department of Education (DoE) WA. The datasets included enrolment details, attendance records, student grades, test scores on the National Assessment Program – Literacy and Numeracy (NAPLAN; an Australian national annual assessment for students in Years 3, 5, 7 and 9 that tests skills across reading, writing, language conventions [spelling, grammar and punctuation] and numeracy), and teacher judgments of students’ Attitude Behaviour and Effort (ABE).

Researchers did not receive information that would identify individual students. The DoE generated a unique identifying number (ID) for each student to protect his/her identity, which was then used by the researchers to match students across the multiple datasets. The SSEN:MMH caters to students from all school sectors in WA (government, Catholic, and independent). However, it only holds data relating to students attending government schools; as a result, students in the Catholic and independent sectors were excluded from analysis. In Western Australia, the Catholic and independent sectors accounted for 30% of primary students and 44% of secondary students in 2016 (Australian Bureau of Statistics, 2017).

A total of 28,697 individual students were in contact with the SSEN:MMH between 2008 and 2016. The current study restricted analyses to students who were supported by the SSEN:MMH in Year 4 and Year 8 during that period. This restriction was due to the schedule of standardised student achievement assessments, which are conducted each year for students in Years 3, 5, 7 and 9. The analysis assessed achievement outcomes after controlling for prior achievement, which excluded Year 3 students from analysis. Achievement outcomes in Year 7 were also excluded due to a policy change in 2015, when Year 7 became the first year of secondary school, instead of Year 8 as in previous years. Year 7 outcomes were, therefore, not comparable over time.

Further, the NAPLAN assessments are administered each year in May, approximately one third into the school year. To ensure the analysis reflected a logical timeline, analyses focused on the progression of two cohorts of students. For the Year 4 cohort, analyses examined prior achievement and behaviour in Semester 1 of Year 3, contact with the SSEN:MMH and related absences across Year 4, and subsequent outcomes in Semester 1 of Year 5. For the Year 8 cohort, analyses examined prior achievement and behaviour in Semester 1 of Year 7, SSEN:MMH contact and absences across Year 8, and subsequent outcomes in Semester 1 of Year 9.

Between 2008 and 2016, a total of 2,199 Year 4 students and 2,160 Year 8 students received support from the SSEN:MMH and were also matched to DoE records. Students needed to have relevant records across three years of school (i.e., Year 3, Year 4, and Year 5) to be included in analysis. Final models for each outcome in Year 4 included between 831 and 1,040 students, and final models for each outcome in Year 8 included between 596 and 793 students.

### Design

The variables in this study included number of teaching support and liaison hours received, days of absence, teacher judgments of ABE, students’ grade point average (GPA), and NAPLAN scores. For analysis, ABE, GPA, and NAPLAN scores represent both predictor variables (i.e., in Years 3 and 7) and outcome variables (Years 5 and 9). Absences and the level of teaching support and liaison provided by the SSEN:MMH relate to Year 4 and Year 8.

#### Teaching and liaison support from the SSEN:MMH

The key variable of interest was the number of support hours provided by the SSEN:MMH. Support hours were recorded as either “teaching” or “liaison” support. Teaching support comprised any direct student contact from teachers on an inpatient ward, through a community-based health program or while convalescing at home. Liaison support comprised any recorded hours of transition support and liaison between SSEN:MMH teachers, the students’ enrolled school, healthcare teams, and families.

#### Absences

Schools are responsible for recording accurate information on student absences. By default, a student is recorded as being present at school when daily records are created. Half-day absences are allocated a code that describes the type of absence, and the codes are updated as further information about the absence comes to hand (e.g., changing from “unexplained” to “vacation”). Information on total half-day absences is aggregated at the semester level. Data included the number of half-days a student was available to attend and the number of authorised and unauthorised half-day absences. The available half-days reflect the number of days a student was enrolled and available to attend school. A typical school year includes up to 380 half-days or 190 full days of school; however, the total can vary slightly from year to year, or for students who change schools during the school year.

Absence rates for the full year were derived by dividing the total number of half-day absences by the total number of half-days available. Transforming half-day absences to an absence rate adjusts for different periods of enrolment between students or between years. To facilitate interpretation throughout analysis, the absence rates were transformed back to reflect the number of full-day absences across the year, where a day of absence could reflect two half-days taken on separate days.

Data relating to the specific codes used to record the reason for student absences were only available from 2013; therefore, we do not assess the educational impacts of different types of absence in this study. However, we do examine a subset of the absence codes to assess how absences are recorded for SSEN:MMH students when they do not attend their enrolled school. When students receive teaching support from the SSEN:MMH, the enrolled school is informed about the nature of the support and provided with instructions about how to record the student’s attendance for that day. Students receiving teaching support are considered to be participating in an approved educational activity, and while they may not be present in the classroom, they are meant to be recorded as participating in an approved program (E-code), which is not considered as an absence.

The E-codes are a potential limitation of the study data because if teaching hours are recorded accurately, classroom absences may be under-counted for students who receive more support. However, SSEN:MMH activities are not the only approved activities under the E-code, as participation in excursions and education extension programs are also included. Therefore, it cannot be assumed that an E-code for a given student relates to the services provided by the SSEN:MMH. An assessment of the use of E-codes for a selection of SSEN:MMH students is provided in the analysis.

#### Attitude, behaviour, and effort (ABE)

ABE assessments were developed by the DoE to include student wellbeing as a dimension of the curriculum to be assessed and reported on. The items were based on the WA Curriculum Framework Core Shared Values, which includes five dimensions: (a) pursuit of knowledge and commitment to achievement of potential, (b) self-acceptance and respect of self, (c) respect and concern for others, (d) social and civic responsibility, and (e) environmental responsibility (School Curriculum and Standards Authority, 2014).

Data from ABE reports were provided for all SSEN:MMH students for all available semesters. For primary students (Year 4), the teacher provides one report each semester. For secondary students (Year 8), up to four teachers provide reports across different subjects (English, Maths, Science, and Society and Environment). The response options were based on the frequency of given behaviours and were not developed as a psychometric scale for research purposes.

The ABE comprises eight statements, including (a) works to the best of their ability, (b) shows self-respect and care, (c) shows courtesy and respect for the rights of others, (d) participates responsibly in social and civic activities, (e) cooperates productively and builds positive relationships with others, (f) is enthusiastic about learning, (g) sets goals and works towards them with perseverance, and (h) shows confidence in making positive choices and decisions.

A previous factor analysis of these items indicated that the items represent a single factor, with very similar factor loadings (Hancock, 2019). Responses to these items were coded as 1 = seldom to 4 = consistently, and then summed across items. For Year 4 students, total scores can range from 8 to 32. For Year 8 students, ABE ratings were summed in each of the four subject areas and then averaged across subjects. As a result, the ABE ratings are not equivalent for Year 4 and Year 8 students.

#### Academic achievement

Student achievement was assessed using both student grades provided by teachers and NAPLAN test scores. Students’ GPA was calculated by taking the average of grades in English, Math, Science, and Humanities & Social Sciences where grades were classified from A = 5 to E = 1.

NAPLAN scores range from approximately zero to 1,000, and scale scores are constructed so that any given score represents the same level of achievement across school years. For the Year 4 cohort, Year 3 scores were used as a control variable and Year 5 scores were assessed as outcome variables. Similarly, for the Year 8 cohort, Year 7 scores were used as control variables and Year 9 scores as the outcome variables.

#### Covariates

In addition to prior educational assessments, the analyses adjusted for gender, Aboriginal and Torres Strait Islander identification, whether students had received prior support from the SSEN:MMH, and school-level advantage. Gender was classified as a binary variable (female/male), as were Aboriginal and Torres Strait Islander identification and prior contact with the SSEN:MMH (yes/no).

School-level advantage was assessed using the Index of Community Socio-Educational Advantage (ICSEA) – a measure developed by the Australian Curriculum, Assessment and Reporting Authority (ACARA) to be able to compare NAPLAN scores of schools that serve statistically similar students. The 2015 measure used for the current study was based on the education and occupation status of parents, the proportion of students in the school who were of Aboriginal or Torres Strait Islander origin, and the accessibility or remoteness of the school. Average ICSEA scores have a mean of 1,000 and a standard deviation of 100. This study converted ICSEA scores to deciles for analysis.

### Procedure

All data processing and analysis was conducted using SAS (version 9.4; SAS Institute Inc., 2016). Analyses were carried out in two stages. First, descriptive analyses examined the level of teaching support and liaison provided to students and how the support varied by level of absence and other student characteristics. A brief analysis also assessed the number of E-codes recorded for students with different levels of teaching support from the SSEN:MMH.

Second, ordinary least-squares regression models were used to estimate student outcomes for each cohort and test the study hypotheses. Regression models were built in two stages. The first model (Model 1) estimated the outcome variables (numeracy, reading, GPA, and ABE) as a function of the number of absences for the cohort year (Year 4, Year 8) and the number of teaching support and liaison hours provided to students that year. Model 1 also included interaction terms between absences and teaching support, and absences and liaison support, to examine the key question of whether the negative association between higher absences and lower outcome scores was lower for students receiving higher levels of support.

Model 2 expanded Model 1 to include prior measures of the outcome variable (e.g., NAPLAN numeracy score in Year 3 when estimating Year 5 numeracy scores), ABE scores, and the list of covariates. That is, for the Year 4 cohort, Model 2 regressed Year 5 academic and behavioural outcomes on Year 3 academic and behaviour variables, the number of absences and teaching and liaison hours provided in Year 4 (and interaction terms), and covariates (gender, school-level advantage, Aboriginal and Torres Strait Islander identification, and prior contact with the SSEN:MMH). For the Year 8 cohort, models regressed Year 9 academic and behavioural outcomes on Year 7 academic and behaviour variables, absences and teaching and liaison hours in Year 8, interaction terms, and covariates. The models did not control for membership of the enrolled school as a small number of students were distributed across schools throughout Western Australia.

## Results

### Student Characteristics and Service Use

**Figure 1:**
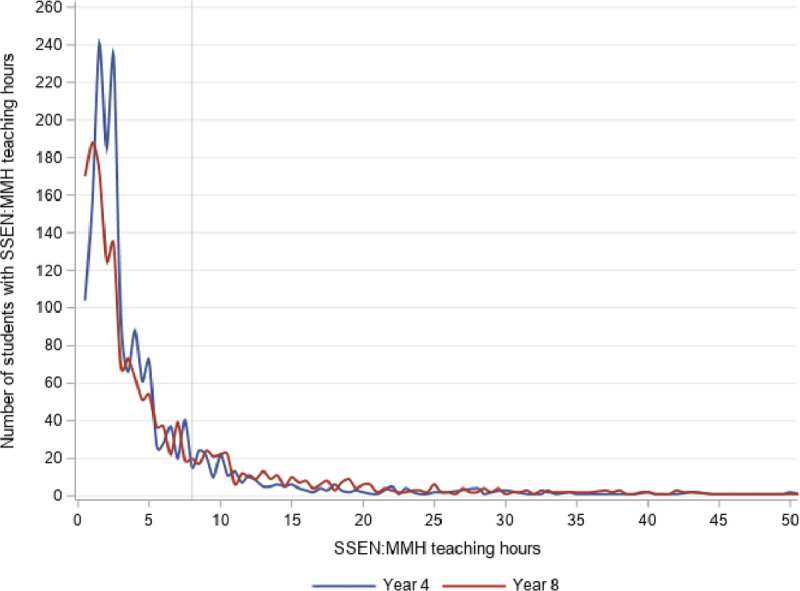
Number of students by SSEN:MMH teaching hours, Year 4 and Year 8. *Note*: Teaching hours were limited to 50 for the purposes of this figure.

**Figure 2:**
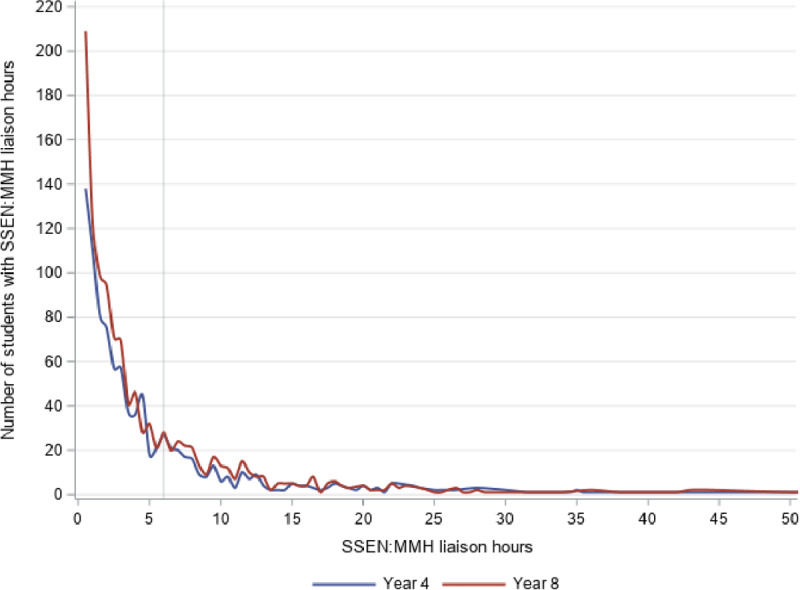
Number of students by SSEN:MMH liaison hours, Year 4 and Year 8. *Note*: Liaison hours were limited to 50 for the purposes of this figure.

Figures 1 and 2 show, respectively, the distribution of teaching (Figure 1) and liaison hours (Figure 2) provided to students across Years 4 and 8. As illustrated in Figure 1, around half of the students coming into contact with the SSEN:MMH received up to 2 hours of teaching support (49.3% Year 4 and 46.4% Year 8). Fewer than one third (32.7% Year 4 and 21.7% Year 8) received between 2 and 4 hours, 6.8% of Year 4 and 6.1% of Year 8 students received between 4 and 8 hours, and a small proportion received more than 8 hours of teaching support (11.2% Year 4 and 15.7% Year 8). Western Australian schools are expected to deliver 25 hours and 50 minutes of instruction per week, and at least 4 hours and 10 minutes on any given school day. Two hours of teaching support, therefore, translates to approximately half a day of regular classroom instruction, 4 hours translates to approximately one day, and 8 hours to approximately two regular school days.

Figure 2 shows that the distribution of liaison hours was as similarly skewed as teaching hours, but fewer hours were provided overall. That is, for Year 4 students, 76% received fewer than 2 hours, 10% between 2 and 4 hours, 7.5% between 4 and 8 hours, and 5.8% more than 8 hours. For Year 8 students, these proportions were 69.5%, 13.9%, 7.9%, and 8.7%, respectively.

The means and standard deviations of NAPLAN scores in Years 3 and 5 for the Year 4 cohort, and in Years 7 and 9 for the Year 8 cohort are provided in Table 1, along with the average test scores for Western Australian students in government schools between 2008 and 2016. Students in contact with the SSEN:MMH had lower numeracy and reading scores both before and after their contact than the average student in their year level across 2008 to 2016, with achievement gaps representing about 0.2 to 0.3 of a standard deviation. Table 2 provides a summary of the student characteristics and the mean number of teaching support and liaison hours provided to students with varying characteristics. For descriptive purposes, continuous variables (e.g., days of absence) have been categorised into groups to show differences in service provision at different levels of those covariates. As noted, large proportions of Year 4 and Year 8 students had missed at least 30 days or about 15% of school days (33.2% of Year 4 students and 62.2% of Year 8 students). Broadly, students who missed more days of school also received more hours of teaching support and liaison, but the increase was not proportional to the increase in absences. For example, Year 4 students who missed up to 10 days received 6.2 hours of teaching support (about 1.5 school days), and students who missed 60 or more days received 11.2 hours on average (about 2.5–3 school days).

**Table 1:** Mean NAPLAN Scale Scores for SSEN:MMH Students and the Broader Student Population in Western Australian government schools, 2008–2016.

	Numeracy		Reading	

	*Mean*	*SD*	*Mean*	*SD*

**Year 4 cohort**				
Year 3	366.9	81.2	372.7	99.2
Year 5	457.9	79.9	460.0	91.7
**Year 8 cohort**				
Year 7	515.9	71.1	558.4	74.4
Year 9	519.0	74.3	555.7	75.5
**WA students 2008–2016**				
Year 3	385.1	77.8	399.3	99.3
Year 5	474.5	73.3	479.9	83.9
Year 7	535.4	73.9	530.7	72.9
Year 9	575.3	73.0	566.3	70.8

**Table 2:** Distribution of Student Characteristics in Year 4 and Year 8, and Mean Number of Teaching Support and Liaison Hours Provided by Student Characteristics.

	Year 4			Year 8		

		Teaching Hours	Liaison Hours		Teaching Hours	Liaison Hours

	*N (%)*	*Mean (SD)*	*Mean (SD)*	*N (%)*	*Mean (SD)*	*Mean (SD)*

**Days of absence in year**						
0 to 9.5	355 (17.4)	6.2 (32.8)	2.4 (6.1)	102 (5.7)	2.2 (6.2)	1.5 (3.8)
10 to 19.5	603 (29.5)	3.9 (15.6)	1.6 (3.9)	268 (15.1)	3.1 (7.2)	1.2 (3.1)
20 to 29.5	408 (19.5)	4.4 (11.9)	1.2 (3.7)	303 (17.1)	3.4 (5.5)	0.9 (2.1)
30 to 39.5	223 (10.9)	3.8 (6.2)	1.4 (3.0)	238 (13.4)	3.4 (5.7)	2.0 (4.2)
40 to 59.5	238 (11.6)	8.9 (30.6)	3.1 (7.0)	300 (16.9)	6.0 (13.4)	2.7 (6.1)
60+	218 (10.7)	11.2 (36.3)	3.9 (8.3)	566 (31.9)	7.9 (18.3)	4.1 (7.2)
**Gender**						
Female	837 (41.0)	6.1 (23.5)	1.4 (4.1)	794 (48.1)	6.5 (15.7)	3.9 (9.7)
Male	1205 (59.0)	5.5 (23.2)	2.5 (6.1)	857 (51.9)	3.9 (9.7)	2.4 (5.3)
**Aboriginal and Torres Strait Islander**						
Yes	324 (15.8)	4.5 (12.6)	1.8 (4.3)	256 (15.4)	6.2 (15.5)	2.1 (5.0)
No	1721 (84.2)	6.0 (24.8)	2.1 (5.5)	1402 (84.6)	5.0 (12.5)	2.5 (5.6)
**Prior contact with SSEN:MMH**						
No	1916 (93.5)	5.9 (24.1)	2.0 (5.4)	1647 (91.8)	5.0 (11.8)	2.5 (5.4)
Yes	134 (6.5)	3.3 (4.8)	2.0 (5.3)	148 (8.2)	6.8 (19.9)	2.3 (5.6)
**School-level disadvantage**						
ICSEA decile 1–3 (more advantaged)	664 (32.6)	5.5 (24.4)	1.6 (4.9)	394 (22.0)	5.1 (15.7)	2.1 (4.8)
ICSEA decile 4–6	524 (25.7)	6.9 (29.5)	2.1 (4.9)	583 (32.5)	5.5 (1.8)	2.6 (5.4)
ICSEA decile 7–8	417(20.5)	5.8 (19.8)	1.9 (4.8)	457 (25.5)	4.4 (10.8)	2.7 (6,4)
ICSEA decile 9–10 (less advantaged)	432 (21.2)	4.8 (15.3)	2.7 (6.8)	358 (20.0)	5.6 (12.3)	2.2 (4.8)
**Prior GPA band**						
1–2 (D–E grade)	253 (15.0)	7.3 (32.8)	3.8 (8.0)	265 (19.4)	4.2 (9.6)	3.5 (6.2)
2.25–3.0 (C–D grade)	779 (46.3)	5.4 (21.3)	2.4 (5.7)	574 (41.9)	4.7 (11.1)	2.5 (5.8)
3.25–4 (B–C grade)	515 (30.6)	4.7 (12.8)	1.1 (3.3)	392 (28.6)	6.6 (17.4)	2.1 (5.3)
4.25+ (A–B grade)	137 (8.1)	5.1 (12.5)	0.6 (2.2)	138 (10.1)	5.3 (9.4)	1.4 (3.0)
**Prior ABE score**						
8–20 (low)	314 (22.5)	6.9 (34.8)	4.8 (7.3)	295 (28.0)	3.7 (8.8)	3.8 (7.1)
21–26	338 (24.2)	4.5 (19.7)	2.5 (6.7)	239 (22.7)	4.9 (9.3)	2.2 (4.2)
27–29	213 (15.2)	4.7 (11.4)	1.0 (2.9)	135 (12.8)	5.7 (11.9)	2.7 (7.4)
30–32 (high)	532 (38.1)	4.4 (10.3)	1.0 (4.1)	383 (36.4)	6.4 (15.2)	1.7 (4.9)

Other notable observations from Table 2 are the over-representation of male students (59%) among Year 4 students, and the over-representation of Aboriginal and Torres Strait Islander students among SSEN:MMH students for both the Year 4 (15.8%) and Year 8 (15.4%) cohorts. Among the broader WA student population in government schools, Aboriginal and Torres Strait Islander students make up 7.6% of the student population (Hancock et al., 2013). Most students in each cohort had no prior contact with the SSEN:MMH (93.5% for Year 4 students 91.8% for Year 8 students).

Table 2 also indicates that students with higher grades or ABE scores in the year before their contact with the SSEN:MMH received fewer liaison hours in Year 4 and 8 than students with lower grades or ABE scores. Besides absence days, student grades, and student ABE, the provision of teaching support and liaison hours did not differ substantially across different student characteristics; however, the means should be interpreted in the context of the very large standard deviations reported.

**Figure 3:**
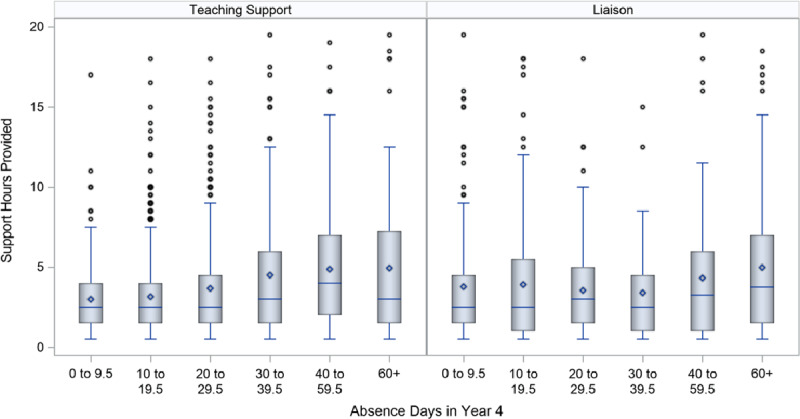
Boxplots of the distribution of teaching support and liaison hours in Year 4, by number of absence days in same year. *Note*: Analysis does not include students with 0 teaching support or liaison hours. Teaching support and liaison hours were limited to 20 for the purposes of this figure.

**Figure 4:**
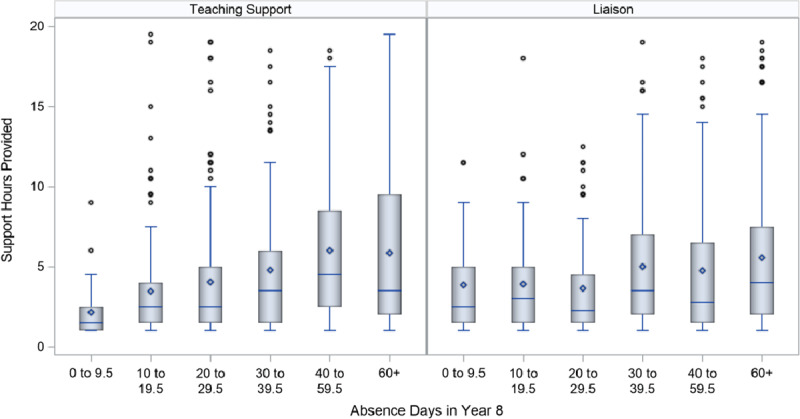
Boxplots of the distribution of teaching support and liaison hours in Year 8, by number of absence days in same year. *Note*: Analysis does not include students with 0 teaching support or liaison hours. Teaching support and liaison hours were limited to 20 for the purposes of this figure.

Further details on the variation in teaching support and liaison hours by level of absence are provided in Figures 3 and 4, which are boxplots summarising the distribution of teaching support and liaison hours at different levels of absences in Year 4 (Figure 3) and Year 8 (Figure 4). For ease of description, the distributions are limited to students with fewer than 20 teaching hours or liaison hours, as Figures 1 and 2 identified very few students above this point. The boxes represent the 25^th^ to 75^th^ percentiles, and the whiskers represent 1.5 times the interquartile range.

Overall, as in Table 2, students who missed more days of school received more teaching support and liaison hours, as evidenced by the increasing length of both the box and the whiskers and the increasing means (dot within the box) and medians (line within the box). However, there were instances of students who were only absent up to 10 days over the year who received as many support hours as students who were absent for more than 40 days of the year, and the increase in means and medians at higher levels of absence were relatively small. More than 50% of Year 4 students with 60 or more absences received less than 8 hours of teaching support and 5 hours of liaison. The boxplots are also characterised by multiple outliers, indicating high levels of variability in the level of support provided.

Absences were also assessed to examine the extent to which absences may be underestimated if schools accurately and routinely apply the E-codes when students are engaged with the SSEN:MMH. The detailed records of a subset of students who were in Years 4 and 8 in 2014 were examined to determine how many half-day E-codes were recorded for students with varying levels of support (see Table 3). The number of half-day N-codes (notified as sick) are also provided for context and comparison. Over two thirds of SSEN:MMH students in Year 4 in 2014 had no E-codes recorded for the year. Of the Year 4 students who received more than 8 hours of teaching support, 73.9% had no E-codes recorded, 13.1% had between 1–10 E-codes, and 13% had more than 20 E-codes. Engagement with the SSEN:MMH is not the only activity for which E-codes can be applied, as students attending class excursions are also considered to be participating in approved educational activities. Therefore, students with very limited support from the SSEN:MMH can still have E-codes recorded from other activities.

**Table 3:** Year 4 Students in 2014: Proportion of Students With E-Codes (Approved Educational Activity) and N-Codes (Notified as Sick) by Level of Teaching Support received in 2014.

	Number of Half-Day E-Codes						Number of Half-Day N-Codes				

		0	1–10	11–20	>20	Total	0	1–10	11–20	>20	Total

	N	%	%	%	%		%	%	%	%	

**Teaching hours in Year 4**											
0 hours	97	69.1	28.9	0.0	2.1	100.0	26.8	43.3	10.3	19.6	100.0
0.5–2 hours	94	78.7	18.1	2.1	1.1	100.0	19.2	33.0	28.7	19.2	100.0
2.5–4 hours	51	82.4	15.7	2.0	0.0	100.0	21.6	31.4	23.5	23.5	100.0
4.5–8 hours	24	83.3	16.7	0.0	0.0	100.0	16.7	12.5	20.8	50.0	100.0
>8 hours	23	73.9	13.0	0.0	13.0	100.0	26.1	17.4	4.4	52.2	100.0
*Total*	289	76.1	20.8	1.0	2.1	100.0	22.5	33.2	19.0	25.3	100.0
**Teaching hours in Year 8**											
0 hours	77	20.8	67.5	11.7	3.9	100.0	19.5	29.9	13.0	37.7	100.0
0.5–2 hours	85	21.2	56.5	20.0	2.4	100.0	12.9	18.8	15.3	52.9	100.0
2.5–4 hours	46	21.7	60.9	10.9	6.5	100.0	19.6	32.6	17.4	30.4	100.0
4.5–8 hours	20	30.0	50.0	15.0	5.0	100.0	15.0	0.0	20.0	65.0	100.0
>8 hours	42	21.4	47.6	9.5	21.4	100.0	11.9	11.9	4.8	71.4	100.0
*Total*	270	21.9	58.5	13.0	6.7	100.0	15.9	21.9	13.7	48.5	100.0

For Year 8 students, a little over 20% had no E-codes, and this pattern was consistent across different levels of teaching support, suggesting that E-codes were more common for secondary students than for primary students in general. Of the Year 8 students, the proportion of students with more than 20 E-codes was substantially higher among students with more than 8 hours of teaching support (21.4%) than for students with fewer support hours (2.0–6.5%).

For both Year 4 and Year 8 students, the association between the number of teaching hours and the number of N-codes was more systematic. That is, for students with progressively higher hours of support progressively higher numbers of N-codes were recorded. Overall, these findings suggest that for the majority of students, schools do not routinely apply the E-codes as instructed by the SSEN:MMH, and the absences are more likely to be recorded as illness. While some level of absence may be underestimated, the absence data are likely to capture most absences from the classroom, even among students receiving higher levels of support.

### Student Outcomes

Tables 4, 5, 6, 7 provide the results from the regression models estimating student outcomes. When examining numeracy and reading outcomes for the Year 4 cohort, the results in Table 4 suggest that before considering prior achievement and other covariates (Model 1), each day of absence in Year 4 was associated with a decrease of 1.10 numeracy points in Year 5 (*p* < .001). Teaching support was not associated with numeracy scores, and each hour of liaison was associated with a decrease of 5.46 numeracy points (*p* < .001). There was no significant interaction between absence days and teaching support hours, but there was a significant interaction between absence days and liaison hours, indicating that the negative association between a higher number of absence days and lower achievement was smaller among students with a higher number of liaison hours (estimate = 0.07, *p* = .001). For example, one liaison hour reduced the negative estimate of absences on achievement by 0.07 points (or 6%), from 1.10 to 1.03, and two hours of liaison reduced the estimate for absences from 1.10 to 0.96. In the context of an assessment with a standard deviation of 80 points, these effect sizes are very small at the level of a day of absence but may be considered as less trivial at higher levels of absences (i.e., for students missing three to four weeks of school). These effects were not statistically significant in Model 2 after adjusting for prior achievement and other covariates, including the effect estimate for higher absences. For the Year 5 NAPLAN models, absences were not associated with achievement after adjusting for prior achievement.

**Table 4:** Students Engaged With the SSEN:MMH in Year 4: Results of Regression Models Estimating Numeracy and Reading Achievement in Year 5.

	Numeracy				Reading			

	Model 1		Model 2		Model 1		Model 2	

	Estimate	*p*-value	Estimate	*p*-value	Estimate	*p*-value	Estimate	*p*-value

Intercept	494.74	<.001	189.41	<.001	496.82	<.001	201.79	<.001
Year 4 absences	–1.10	<.001	–0.18	.093	–1.01	<.001	–0.07	.575
Teaching hours	0.56	.039	0.22	.201	0.35	.269	–0.20	.343
Absence days × Teaching hours interaction	0.01	.248	0.01	.848	0.01	.163	0.00	.616
Liaison Hours	–5.46	<.001	–1.00	.211	–5.83	<.001	0.46	.627
Absence days × Liaison hours interaction	0.07	.001	0.01	.645	0.09	<.001	0.00	.958
Year 3 achievement			0.71	< 001			0.65	<.001
Year 3 ABE score			1.13	<.001			1.56	<.001
School-level advantage			–1.62	.011			–1.44	.068
Female vs male			–8.04	.025			–3.80	.383
Aboriginal vs non-Aboriginal			–6.55	.225			–16.77	.011
Prior contact with SSEN:MMH (no vs yes)			4.31	.520			–9.98	.227
*N* in model	833		831		845		843	
R ^2^	0.08		0.65		0.05		0.57	

*Note*: Model 1 estimates are restricted to students who also had previous achievement and ABE scores.

**Table 5:** Students Engaged With the SSEN:MMH in Year 8: Results of Regression Models Estimating Numeracy and Reading Achievement in Year 9.

	Numeracy				Reading			

	Model 1		Model 2		Model 1		Model 2	

	Estimate	*p*-value	Estimate	*p*-value	Estimate	*p*-value	Estimate	*p*-value

Intercept	592.93	<.001	164.5	<.001	583.54	<.001	193.04	<.001
Year 8 absences	–0.63	<.001	–0.17	.029	–0.51	<.001	–0.04	.652
Teaching hours	–0.03	.974	–0.62	.167	–0.08	.921	–0.46	.371
Absence days × Teaching hours interaction	0.00	.815	0.01	.251	0.00	.796	0.01	.421
Liaison Hours	–6.79	<.001	–2.04	.021	–4.57	.003	–1.19	.249
Absence days × Liaison hours interaction	0.09	<.001	0.03	.035	0.07	.016	0.00	.914
Year 7 achievement			0.79	<.001			0.68	<.001
Year 7 ABE score			0.65	.036			1.02	.003
School-level advantage			–2.02	.003			–2.45	<.001
Female vs male			–3.12	.360			6.37	.091
Aboriginal vs non-Aboriginal			–0.74	.894			–8.39	.190
Prior contact with SSEN:MMH (no vs. yes)			–7.69	.204			–0.88	.895
*N*	664		596		672		605	
*R* ^2^	0.06		0.72		0.03		0.62	

**Table 6:** Students Engaged With the SSEN:MMH in Year 4: Results of Regression Models Estimating GPA and ABE Scores in Year 5.

	GPA				ABE Total			

	Model 1		Model 2		Model 1		Model 2	

	Estimate	*p*-value	Estimate	*p*-value	Estimate	*p*-value	Estimate	*p*-value

Intercept	3.272	<.001	0.830	<.001	27.97	<.001	12.28	<.001
Year 4 absences	–0.013	<.001	–0.003	<.001	–0.06	<.001	–0.01	.444
Teaching hours	0.006	.006	0.001	.312	0.06	.002	0.01	.502
Absence days × Teaching hours interaction	0.000	.165	0.000	.630	0.00	.432	0.00	.661
Liaison Hours	–0.060	<.001	–0.014	.007	–0.60	<.001	–0.18	<.001
Absence days × Liaison hours interaction	0.001	<.001	0.001	.011	0.01	<.001	0.00	.123
Year 3 GPA			0.691	<.001			1.02	<.001
Year 3 ABE score			0.011	<.001			0.49	<.001
School-level advantage			–0.034	<.001			–0.26	<.001
Female vs male			0.045	.121			1.85	<.001
Aboriginal vs non-Aboriginal			–0.037	.381			–0.34	.425
Prior contact with SSEN:MMH (no vs yes)			–0.025	.641			–1.03	.060
*N*	1,041		1,037		1,045		1,040	
R^2^	0.14		0.69		0.12		0.49	

*Note*: GPA scores are reported to three decimal places due to scaling of the variable.

**Table 7:** Students Engaged With the SSEN:MMH in Year 8: Results of Regression Models Estimating GPA and ABE Scores in Year 9.

	GPA				ABE Total			

	Model 1		Model 2		Model 1		Model 2	

	Estimate	*p*-value	Estimate	*p*-value	Estimate	*p*-value	Estimate	*p*-value

Intercept	3.44	<.001	0.671	<.001	22.66	<.001	10.56	<.001
Year 8 absences	–0.016	<.001	–0.009	<.001	–0.09	<.001	–0.06	<.001
Teaching hours	0.008	.297	0.003	.650	0.11	.044	0.05	.259
Absence days × Teaching hours interaction	0.000	.557	0.000	.704	0.00	.519	0.00	.601
Liaison Hours	–0.071	<.001	–0.016	.130	–0.34	<.001	–0.06	.424
Absence days × Liaison hours interaction	0.001	<.001	0.000	.294	0.003	.008	0.00	.810
Year 7 GPA			0.687	<.001			1.12	.001
Year 7 ABE score			0.018	.001			0.33	<.001
School-level advantage			–0.021	.055			–0.01	.870
Female vs male			0.068	.225			0.51	.244
Aboriginal vs non-Aboriginal			–0.158	.065			–1.28	.053
Prior contact with SSEN:MMH (no vs yes)			–0.025	.797			–1.01	.178
*N*	869		793		764		694	
*R* ^2^	0.23		0.56		0.22		0.36	

*Note*: GPA scores are reported to three decimal places due to scaling of the variable.

For Year 9 NAPLAN outcomes (Table 5), the results of Model 1 indicated lower numeracy achievement for each day of absence (estimate = –0.63, *p* < .001) and each liaison hour (estimate = –6.79, *p* < .001), and significant liaison hours by absence interaction (estimate = 0.09, *p* < .001). These effect sizes halved but remained statistically significant after controlling for prior academic and behavioural measures and covariates. No statistically significant effects were observed for Year 9 reading outcomes, including for higher absences. For both numeracy and reading, higher levels of prior achievement, ABE scores, and school-level advantage were all associated with higher achievement in Year 9.

For GPA outcomes, the adjusted model for Year 5 students (Table 6, Model 2) showed that higher numbers of absences and liaison hours were associated with lower GPA scores (absence estimate = –0.003, *p* < .001; liaison estimate = –0.014, *p* = .007); in addition, there was a significant interaction suggesting that the negative association between higher absences and lower GPA scores was reduced by 0.001 (*p* = .011) for each additional liaison hour in Year 4; but, again, the effect sizes were very small. For Year 9 outcomes (Table 7), teaching support and liaison hours in Year 8 were not associated with GPA scores.

For ABE outcomes, Table 6 shows that the adjusted model for Year 5 students (Model 2) found that higher numbers of liaison hours in Year 4 were associated with lower ABE scores in Year 5 (estimate = –0.18, *p* < .001), but absences and teaching support were not associated with ABE outcomes after controlling for prior ABE and other covariates. For Year 9 students, Table 7, Model 2 shows that higher numbers of absences in Year 8 were associated with lower ABE scores in Year 9 (estimate = –0.06, *p* < .001), but teaching hours and liaison hours were not associated with ABE outcomes.

## Discussion

The SSEN:MMH provides an important service for students with medical and mental health conditions. The school recognises that all students have a right to participate in education (United Nations, 1989) and that school absences caused by medical and mental health conditions can severely impact students’ ability to remain engaged with their education. This study examined the service provision of the SSEN:MMH and the extent to which teaching and liaison support mitigated the impacts of missing school. To examine these relationships, the study used longitudinal administrative education records of students engaged with the SSEN:MMH and analytic methods that adjusted for key baseline characteristics (e.g., prior achievement) to reduce bias.

There were two findings of note. First, while higher levels of teaching support in Year 4 were associated with marginally higher numeracy scores in Year 5, this association was explained by other factors, mainly prior achievement level, suggesting that higher achieving students are more likely to opt into receiving educational support from the SSEN:MMH, and also more likely to receive more hours of support. In all other models, higher levels of teaching support were not associated with student outcomes, and there was no evidence that higher levels of teaching support reduced the negative association between higher absences and lower outcomes. However, these null findings must be considered in terms of the broader descriptive findings on the level of support that students were provided. Thus, the descriptive analysis of teaching hours suggests that any benefits associated with this support are very difficult to identify in the administrative data examined. For example, many students with very high levels of absence, more than 40 days in a year, received teaching support that was less than the equivalent of two days of school.

Two days of teaching support in the context of missing 40 or more days of school may not seem an adequate amount of support to mitigate those absences. However, while it could not be examined in the data provided, students can spend time at home both before and after their hospitalisation, and not all of their absences would relate to their condition. Additionally, previous research suggests that at a population level, illness accounts for approximately 60% of absences (Rothman, 2002), and that students with higher rates of absence due to illness have as many absences for other reasons as other students (Hancock, 2019). Therefore, it is unreasonable to expect that students who miss 5–6 times as many school days as other students receive 5–6 times as many teaching hours to mitigate those absences.

When considering that the SSEN:MMH provides the majority of its support to students within hospital settings, and that students miss school for many different reasons, it is perhaps not surprising to learn that there was no significant association between teaching support and students’ outcomes based on the measures assessed. It is also possible that given the hours of support provided and the range of possible scores on the outcome variables, the level of support provided contributed to positive outcomes that could not be detected using the measures available. Overall, these data highlight the challenge that the SSEN:MMH faces in mitigating the effect of missing school, mainly, that most students miss more school than the SSEN:MMH can reasonably respond to in the time that students are in their settings. Instead, the support of students’ enrolled schools on an ongoing basis is essential for these students to overcome the educational obstacles posed by their medical and mental health condition.

The second related finding was that while students with higher levels of liaison support in Years 4 and 8 had lower outcomes overall in Years 5 and 9, significant interaction terms suggested that higher numbers of liaison hours reduced the negative association between higher absences and lower GPA scores in Year 5, and lower numeracy scores in Year 9. These interaction effects, while small and inconsistent, provide preliminary evidence that the connections that the SSEN:MMH strengthens between students, families, schools, and health professionals may help students to remain engaged with their school. As opposed to teaching supports, which provide direct instruction to students, liaison services can help teaching staff in students’ enrolled schools to understand medical and mental health conditions and the types of supports that may improve a given student’s engagement. Based on these results, liaison services may, therefore, have a more enduring impact on student outcomes than was found for teaching support.

As noted, there were also statistically significant effects suggesting that a higher number of liaison hours was associated with *lower* achievement and ABE scores in the following year; however, this pattern was likely an artefact of the more complex needs of students who require additional liaison support. The descriptive data showed that in the year prior to contact with the SSEN:MMH, students with lower grades and ABE scores received more hours of liaison support than students with higher grades and ABE scores, suggesting that students who were already struggling at school had a higher need for liaison support than other students. Higher numbers of liaison hours may also reflect increased challenges in communicating with the enrolled school, which may itself be associated with challenges with the quality of educational interventions and relationships within that enrolled school, or problems in the relationship between the student, his/her family, and the enrolled school. As such, the negative effects observed across educational and behavioural outcomes in this study are likely to reflect pre-existing challenges rather than deficits in the liaison processes within the SSEN:MMH.

The overall nonsignificant effects may also relate to several factors associated with the sensitivity of available administrative data and variables included in analysis. SSEN:MMH students are a heterogeneous group with medical and mental health conditions ranging from simple and short-term to complex and severe. While many relevant control variables were included in the models, these variables could not account for the severity of students’ conditions. The severity of students’ conditions, and comorbidities in particular, clearly has an impact on educational and behavioural outcomes in the year following contact with the SSEN:MMH, and variables available within the current analysis may not sufficiently account for this variability. For example, secondary students may face additional barriers in their transition back to their enrolled school where they have multiple classes, teachers, and peer groups, particularly with regard to socio-emotional and mental health-related conditions that require more extensive liaison services. Qualitative analysis integrating case studies and interview data may more adequately acknowledge and address these barriers in future research. Furthermore, while the objectives of liaison within the SSEN:MMH encompass educational and behavioural outcomes, many key objectives centre around improved communication with enrolled schools, increased professional education of staff within enrolled schools, and ensuring students are well supported in their transition into and out of hospital school. Outcomes that measure these objectives were not collected within the standard administrative datasets used for this study. Additionally, while measures related to educational engagement and social support were not a focus of this study, it is likely that peer-to-peer learning activities occurring within SSEN:MMH, such as peer tutoring or cooperative learning – which are not captured by measures of teaching support – can support continued educational engagement as well as maintaining social relationships and wellbeing for students disconnected from regular schooling while in hospital. These activities can enrich teaching support and potentially increase hours of learning without any additional cost.

Finally, the key variables of interest were the interaction terms that assessed if the negative association between higher absences and lower outcomes was mitigated by higher teaching and liaison hours. This approach assumes that students have lower scores if they miss more days of school. However, while higher numbers of absences were significantly associated with lower outcomes in all the unadjusted models, the association was significant only for four of the eight outcomes considered (Year 9 numeracy, Year 5 and 9 GPA, and Year 9 ABE) following adjustment for baseline variables. Previous research has shown that the negative effect of absences is more strongly related to numeracy skills than literacy skills (Gottfried & Kirksey, 2017; Hancock, 2019), so the finding that absences were not associated with reading outcomes is somewhat consistent with the broader literature. It is not clear why higher absences in Year 4 were not associated with lower Year 5 numeracy scores or Year 5 ABE scores given previous research. However, if there is no effect of absence on subsequent outcomes, it is difficult to argue that teaching or liaison support will help to reduce those effects.

## Conclusion

This study was not able to provide evidence that teaching support reduces the negative impact of higher numbers of absences for students with medical and mental health needs. However, the study does provide some evidence that the number of liaison support hours may mitigate the association between missing more school and education or behavioural outcomes the following year. The results highlighted the challenges faced by the SSEN:MMH in terms of the broader absence patterns of students and the opportunity they have to impact on the negative effects of missing school. Overall, the effects observed in this analysis highlight the complexity of student experiences prior to, during, and following hospital school support and the challenges of ongoing and evolving data collection within a system designed to privilege support for vulnerable children and adolescents over data administration.

### Limits of the Study

The regression models were restricted by the variables available in the datasets used in the analysis, and did not include additional factors that may have more adequately predicted students’ educational and behavioural outcomes. The severity of students’ conditions, and comorbidities in particular, has an impact on educational and behavioural outcomes in the year following contact with the SSEN:MMH, and variables available within the current analysis may not have sufficiently accounted for this factor.

Small sample size in population subgroups identified within the SSEN:MMH dataset precluded the analysis of these subgroups (e.g., students in the orthopaedic ward or students receiving community-based diabetes support). This limitation meant we were unable to analyse or report on whether teaching or liaison support hours are more effective when directed specifically at these subgroups.

We used teacher ratings of attitude, behaviour, and effort to estimate the effects of SSEN:MMH on student’s behavioural outcomes. These ratings may be biased by enrolled school teachers’ perceptions of student behaviour, particularly if students have been absent for prolonged periods due to their health condition.

The procedures for recording attendance and absences in enrolled schools and inconsistent record management by schools also meant that for some students, the absence measure may not accurately reflect the number of days of school they were absent from the classroom. If records were managed as intended, the hours of teaching support provided to students would be recorded as an approved educational activity, and not as absence. While the results suggested that in many cases the records were not updated to reflect these activities as they should have been, the level of classroom absence may be underestimated for some students, which in turn may affect the estimates relating to the effects of absence on student outcomes.

### Implications for Further Research

Students with complex and/or severe educational needs should ideally be identified to help understand the circumstances of students over and above the level of engagement these students have with the SSEN:MMH. One way to better understand and measure case complexity is to develop an index that rates the severity of students’ medical and/or mental health conditions with reference to the potential impacts of the conditions on educational attainment. Such an index should be developed in consultation with content-area experts from the SSEN:MMH (or equivalent institution) and based on complexity of condition/s, co-morbidities, inpatient/outpatient contact, and any other factors deemed relevant.

Additionally, school characteristics likely to impact on educational and behavioural outcomes should be integrated into future analysis where possible; for example, quality of communication, supportive attitudes of teachers, and engagement with professional learning.

Further, case studies and interview data could be included into future study designs to acknowledge the unique barriers to educational achievement faced by adolescents experiencing long-term, chronic and/or comorbid health conditions. Qualitative data gained from these methods could assist in the development of variables and models specific to this cohort.

Liaison activities have been identified as particularly effective for improving educational outcomes in students with chronic health conditions where these activities improve communication between healthcare teams and educators from student’s enrolled schools. Outcomes that integrate measures directly related to liaison activities should be developed for future studies informed by internal evaluative activities of hospital school programs, including qualitative data. Such outcomes would assist in accounting for the critical role these processes play in improving educational attainment and behaviour for students with chronic health conditions.
